# Frequency, Antimicrobial Susceptibility, and Molecular Characterization of Carbapenem-Resistant Enterobacterales Stratified by United States Census Divisions: Results From the INFORM Program (2018–2022)

**DOI:** 10.1093/ofid/ofaf005

**Published:** 2025-04-11

**Authors:** Helio S Sader, John H Kimbrough, Timothy B Doyle, Marisa L Winkler, Mariana Castanheira

**Affiliations:** Element Iowa City (JMI Laboratories), North Liberty, Iowa, USA; Element Iowa City (JMI Laboratories), North Liberty, Iowa, USA; Element Iowa City (JMI Laboratories), North Liberty, Iowa, USA; Element Iowa City (JMI Laboratories), North Liberty, Iowa, USA; Element Iowa City (JMI Laboratories), North Liberty, Iowa, USA

**Keywords:** CRE, KPC, metallo-beta-lactamase, NDM, OXA-48

## Abstract

**Background:**

Recently approved β-lactamase inhibitor combinations, such as ceftazidime-avibactam, meropenem-vaborbactam, and imipenem-relebactam, have demonstrated a broad spectrum of activity against carbapenem-resistant Enterobacterales (CRE) from US hospitals, but resistance may emerge with the increasing use of these compounds. Aztreonam-avibactam was recently approved in Europe and it is under clinical development in the United States. We evaluated the activity of aztreonam-avibactam and comparators against CREs from US hospitals.

**Methods:**

A total of 45 497 Enterobacterales isolates were consecutively collected from 79 US medical centers (36 states) and susceptibility tested by broth microdilution. Aztreonam-avibactam was tested with avibactam at a fixed 4 mg/L and a susceptible breakpoint of ≤4 mg/L was applied for comparison. CRE isolates were screened for carbapenemase by whole-genome sequencing.

**Results:**

Aztreonam-avibactam inhibited >99.9% of Enterobacterales at ≤4 mg/L. CRE frequencies varied from 0.2% (New England) to 2.4% (Middle Atlantic). Aztreonam-avibactam was active (minimum inhibitory concentration ≤4 mg/L) against 98.6% (408/414) of CREs overall, whereas susceptibility to ceftazidime-avibactam and meropenem-vaborbactam were lowest in the Mountain division (67.7% and 74.2%, respectively) and highest (100.0%) in West North Central. *Klebsiella pneumoniae* carbapenemase was the most common carbapenemase (65.5% of CREs), followed by New Delhi MBL (10.6%) and oxacillinase-48–like (2.7%). The occurrence of *Klebsiella pneumoniae* carbapenemase among CREs varied from 14.3% (New England) to 77.8% (East South Central), whereas the frequency of MBLs ranged from ≤3.0% (4 divisions) to 19.4% in Mountain and 42.9% in New England.

**Conclusions:**

Aztreonam-avibactam showed potent activity against CRE, including MBL producers. Resistance to ceftazidime-avibactam and meropenem-vaborbactam was observed among CRE because of increasing occurrence of MBL-producing isolates.

Infections caused by carbapenem-resistant Enterobacterales (CRE) are linked to greater probability of death than infections caused by carbapenem-susceptible Enterobacterales, and the impact of carbapenem resistance on mortality is mostly related to the delay before administering effective antimicrobial therapy [1–3].

Recently available β-lactamase inhibitor combination agents such as ceftazidime-avibactam, meropenem-vaborbactam, and imipenem-relebactam represented a significant improvement in the treatment of CRE infections, especially with *Klebsiella pneumoniae* carbapenemase (KPC)-CRE [4, 5]. However, meropenem-vaborbactam and imipenem-relebactam have limited activity against oxacillinase (OXA)-48–like producers and none of these newer β-lactamase inhibitor combination agents are active against the metallo-β-lactamases (MBLs), including the New Delhi MBL (NDM) [6, 7].

Cefiderocol represented another important addition to the armamentarium for treatment of CRE infections. However, cefiderocol minimum inhibitory concentrations (MICs) may increase to resistance level when the organism produces some β-lactamases as seen mainly in the NDM types NDM-1, -5, -7, and -9; *Pseudomonas*-extended-resistant (PER) types PER-1, -6, and -7; KPC variants conferring resistance to ceftazidime-avibactam, OXA-427 [8]; and Verona integron–encoded MBL (VIM-1) [8–10].

The production of carbapenemase represents the foremost mechanism of carbapenem resistance among Enterobacterales. Globally, common carbapenemases in Enterobacterales include KPC, OXA-48–like β-lactamases, MBLs such as NDM, the “active in imipenem” family of carbapenemases, and Verona integron–encoded MBLs (VIM). Notably, carbapenemase distributions are regional. KPC enzymes predominate in the Americas, Italy, Israel, Greece, and Portugal; OXA-48–like enzymes are the most common carbapenemases in much of Europe (except for Italy, Greece, and Portugal), the Middle East (except Israel), and North Africa; and NDM types are the most prevalent carbapenemase in South Asia [11].

The epidemiology of carbapenemases is very dynamic and appears to be changing in the United States in the past few years. KPC producers used to account for the vast majority of CREs in US medical centers, but a progressive increase in the frequency CRE carrying other carbapenemases, mainly NDM types, has been observed more recently which may compromise the spectrum of activity of these recently approved β-lactamase inhibitor combination agents and cefiderocol against CRE [7, 12].

Aztreonam-avibactam is under clinical development for treatment of infections caused by Gram-negative bacteria, including MBL producers, and it has been approved (April 2024) by the European Medicines Agency in the European Union (https://www.ema.europa.eu/en/news/new-antibiotic-fight-infections-caused-multidrug-resistant-bacteria; accessed on 1 July 2024). We have previously analyzed the activity of aztreonam-avibactam against isolates from other geographic regions (eg, United States) and against selected subsets of isolates from US hospitals [13–15]. Moreover, in this investigation, we performed a comprehensive evaluation of the antimicrobial susceptibility of Enterobacterales and CRE isolates and the epidemiology of carbapenemases in the United States and stratified the results by US Census Division.

## MATERIALS AND METHODS

### Organism Collection

Bacterial isolates were collected via a network of medical sites participating in the International Network for Optimal Resistance Monitoring (INFORM) Surveillance Program and sent to Element Iowa City (JMI Laboratories; North Liberty, IA, USA) for susceptibility testing [16]. Each participating center was requested to collect a specific number of consecutive bacterial isolates each year from patients hospitalized with the following infection types: bloodstream infection, pneumonia, urinary tract infection, skin and skin structure infection, and intra-abdominal infection. The number of isolates varied per infection type. Only isolates determined to be the probable cause of infection by local criteria were included in the investigation.

**Figure 1.**
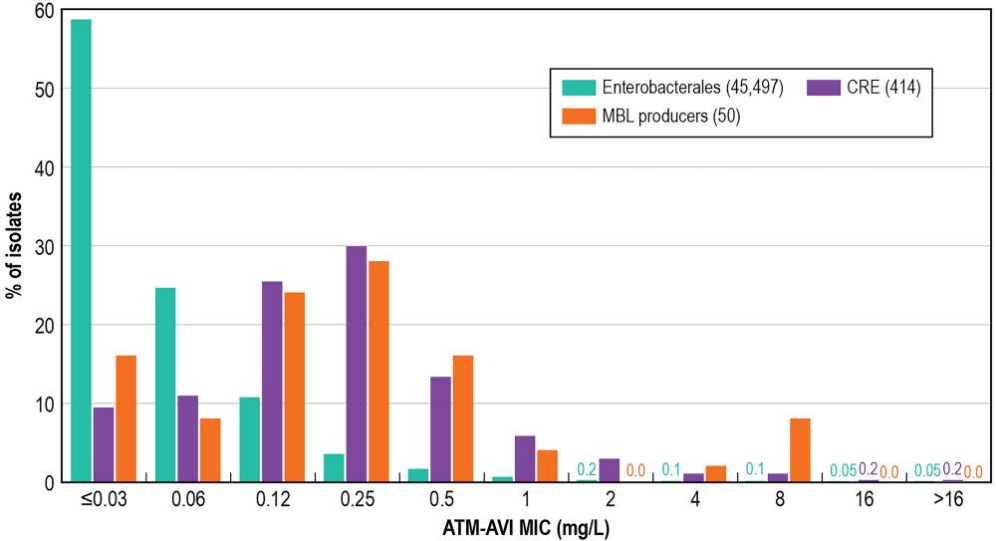
Aztreonam-avibactam (ATM-AVI) minimum inhibitory concentration (MIC) distributions for Enterobacterales and carbapenem-resistant Enterobacterales (CRE) isolates from US medical centers (2018–2022).

A total of 45 497 Enterobacterales isolates were consecutively collected from 79 US medical centers (36 states) in 2018–2022 and susceptibility results were stratified by US Census Divisions (Figure 1). The number of participant centers per Census Division varied from 5 (Census Division 1 [New England]) to 13 (Census Division 3 [East North Central]). Species identification was confirmed by MALDI Biotyper (Bruker Daltonics, Billerica, MA, USA), standard biochemical tests, and/or genome sequencing, when necessary. CRE was defined as any isolate displaying MIC values of ≥4 mg/L for imipenem and/or meropenem. Imipenem was not applied for *Proteus mirabilis* or indole-positive Proteeae due to their intrinsically elevated MIC values.

### Susceptibility Testing

All isolates were susceptibility tested by the reference broth microdilution method specified by CLSI standards [17]. Ceftazidime-avibactam, ceftolozane-tazobactam, imipenem-relebactam, and piperacillin-tazobactam were tested with a β-lactamase inhibitor at a fixed concentration of 4 mg/L; meropenem-vaborbactam was tested with vaborbactam at a fixed concentration of 8 mg/L. All tests were conducted in a central monitoring laboratory (Element Iowa City [JMI Laboratories]). Cefiderocol was tested only against CRE isolates on iron-depleted media [17].

The aztreonam breakpoint published by CLSI (≤4 mg/L) was applied for aztreonam-avibactam for comparison [17]. CLSI and US Food and Drug Administration breakpoints (https://www.fda.gov/drugs/development-resources/antibacterial-susceptibility-test-interpretive-criteria) were applied to the comparator agents where available [18]. Concurrent quality control testing was performed to ensure proper test conditions and procedures.

### β-Lactamase Screening

All CRE isolates (n = 414) were tested for β-lactamase–encoding genes using next-generation sequencing. Total genomic DNA was extracted using the fully automated Thermo Scientific KingFisher Flex Magnetic Particle Processor (Cleveland, OH, USA). DNA extracts were quantified using the Qubit High Sensitivity DS-DNA assay (Invitrogen, ThermoFisher Inc.) and normalized to 0.2 ng/µL. A total of 1 ng high-quality genomic DNA was used as input material for library construction using the Nextera XT DNA library preparation kit (Illumina, San Diego, CA, USA). Libraries were normalized using the bead-based normalization procedure (Illumina) and sequenced on MiSeq. The generated FASTQ files were assembled using SPAdes Assembler and subjected to proprietary software (Element Iowa City [JMI Laboratories]) for screening of β-lactamase genes [19]. An in-house proprietary bioinformatic pipeline and an Element Iowa City–curated resistance gene database (Version 3; uses Python v2.7.9, SPAdes v3.15.3, and BBMap v36.x) based on the NCBI Bacterial Antimicrobial Resistance Reference Gene Database (https://www.ncbi.nlm.nih.gov/bioproject/PRJNA313047) was used for the in silico analysis [20].

## RESULTS

The antimicrobial susceptibility of the entire Enterobacterales collection (n = 45 497) and the CRE isolates (n = 414) is shown in Table 1 stratified by US Census Division. Aztreonam-avibactam was the most active compound (MIC_50/90_, ≤0.03/0.12 mg/L) and inhibited >99.9% of isolates at ≤4 mg/L. Only 20 isolates showed aztreonam-avibactam MIC values >4 mg/L: 16 isolates at 8 mg/L, 2 isolates at 16 mg/L, and 2 isolates at >16 mg/L (Table 1 and Figure 1). Ceftazidime-avibactam (MIC_50/90_, 0.12/0.25 mg/L; 99.8% susceptible) and meropenem-vaborbactam (MIC_50/90_, 0.03/0.06 mg/L; 99.8% susceptible) were also highly active against Enterobacterales (Table 1). Meropenem (MIC_50/90_, 0.03/0.06 mg/L) was active against 99.0% of isolates, whereas imipenem-relebactam (MIC_50/90_, 0.12/1 mg/L; 92.4% susceptible), ceftolozane-tazobactam (MIC_50/90_, 0.25/1 mg/L; 94.6% susceptible), and piperacillin-tazobactam (MIC_50/90_, 2/16 mg/L; 89.1% susceptible) were less active than the other β-lactamase inhibitor combinations (Table 1). Among non–β-lactam agents, the most active compounds were tigecycline (MIC_50/90_, 0.25/2 mg/L; 95.2% susceptible per US Food and Drug Administration) and amikacin (MIC_50/90_, 2/4 mg/L; 95.0% susceptible; Table 1).

**Table 1.** Antimicrobial Susceptibility of Organisms Stratified by US Census Divisions (2018–2022)

Antimicrobial Agent	% Susceptible per Census Division^a^ (No. of Isolates)									
	1	2	3	4	5	6	7	8	9	All
Enterobacterales	(3294)	(7077)	(7799)	(4216)	(6160)	(3347)	(4444)	(4059)	(5101)	(45 497)
Aztreonam-avibactam^b^	100.0	99.9	99.9	>99.9	>99.9	>99.9	99.9	>99.9	>99.9	>99.9
Ceftazidime-avibactam	99.9	99.5	>99.9	100.0	99.9	99.9	99.9	99.7	99.9	99.8
Meropenem-vaborbactam	>99.9	99.4	>99.9	100.0	99.9	100.0	99.7	99.7	99.8	99.8
Imipenem-relebactam^c^	92.7	90.9	92.1	93.2	94.0	92.0	91.9	93.6	92.2	92.4
Ceftolozane-tazobactam	96.5	91.5	95.8	96.2	95.0	95.2	94.1	94.1	94.6	94.6
Piperacillin-tazobactam^d^	92.8	84.1	91.0	92.3	89.3	90.0	86.6	89.3	89.3	89.1
Ceftriaxone	87.8	74.3	87.2	88.7	84.8	84.1	80.3	83.9	82.4	83.3
Ceftazidime	90.2	79.9	90.1	91.7	87.8	88.0	84.8	87.4	86.3	87.0
Cefepime^d^	92.8	82.7	93.4	94.8	91.2	91.0	87.4	91.2	89.9	90.2
Meropenem	99.7	97.5	99.4	99.8	99.5	99.5	98.4	99.3	99.0	99.0
Imipenem^c^	92.5	88.4	90.1	92.0	91.9	90.2	90.5	91.4	90.1	90.6
Levofloxacin	85.0	76.1	84.9	87.2	82.4	81.6	76.9	85.5	84.2	82.4
Gentamicin	93.1	88.5	93.1	94.2	91.8	92.7	89.5	92.7	91.4	91.7
Amikacin	95.7	93.2	95.4	96.0	95.4	95.6	93.1	95.8	95.5	95.0
Tigecycline^e^	96.5	94.2	94.8	96.5	95.6	94.9	94.7	95.9	94.8	95.2
CRE	(7)	(170)	(43)	(5)	(36)	(18)	(60)	(31)	(44)	(414)
Aztreonam-avibactam^b^	100.0	99.4	97.7	100.0	100.0	94.4	96.7	100.0	97.7	98.6
Ceftazidime-avibactam	71.4	83.5	97.7	100.0	91.7	83.3	93.3	67.7	93.2	87.0
Meropenem-vaborbactam	85.7	80.0	95.3	100.0	91.7	100.0	83.3	74.2	86.4	84.5
Imipenem-relebactam^c^	57.1	77.1	86.0	100.0	80.6	94.4	83.3	67.7	79.5	79.5
Cefiderocol	71.4	92.4	95.3	80.0	97.2	88.9	96.7	100.0	97.7	94.2
Levofloxacin	28.6	23.7	23.3	0.0	25.0	50.0	21.7	41.9	36.4	27.1
Gentamicin	100.0	45.9	65.1	80.0	61.1	33.3	55.0	74.2	61.4	55.1
Amikacin	100.0	55.3	48.8	60.0	75.0	55.6	80.0	64.5	54.5	61.4
Tigecycline^e^	85.7	94.7	95.3	100.0	97.2	100.0	93.3	96.8	93.2	94.9

^a^Census Divisions: 1, New England; 2, Middle Atlantic; 3, East North Central; 4, West North Central; 5, South Atlantic; 6, East South Central; 7, West South Central; 8, Mountain; 9, Pacific.

^b^% inhibited at ≤8 mg/L.

^c^All Enterobacterales species were included in the analysis, but CLSI excludes *Morganella*, *Proteus*, and *Providencia* species.

^d^Indicates percentage of susceptible; does not include susceptible dose dependent.

^e^US Food and Drug Administration–susceptible breakpoint of ≤2 mg/L was applied.

The activity of aztreonam-avibactam against Enterobacterales was very similar among all Census Divisions with 99.9% to 100.0% of isolates inhibited at ≤4 mg/L (Table 1). Enterobacterales susceptibility rates for all other agents tested except amikacin were the lowest in Census Division 2 (Middle Atlantic), which had participant centers in the states of New York (4 centers), New Jersey (3), and Pennsylvania (2). The Census Division with the second lowest susceptibility rates in general was Census Division 7 (West North Central), which included participant centers in Texas (5 centers), Arkansas (1), and Louisiana (1). The highest susceptibility rates were generally observed in Census Division 4 (West North Central), which included centers in Iowa (2 centers), Kansas (1), Minnesota (2), Missouri (2), North Dakota (1), and Nebraska (1) (Table 1 and Supplementary Figure 1).

**Figure 2.**
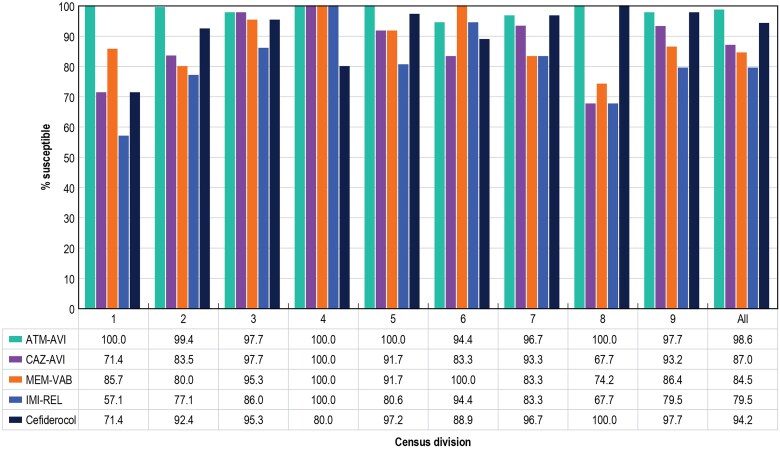
Activities of aztreonam-avibactam (ATM-AVI), ceftazidime-avibactam (CAZ-AVI), meropenem-vaborbactam (MEM-VAB), imipenem-relebactam (IMI-REL), and cefiderocol against carbapenem-resistant Enterobacterales (CRE) isolates from US medical centers (2018–2022). Census Divisions: 1, New England; 2, Middle Atlantic; 3, East North Central; 4, West North Central; 5, South Atlantic; 6, East South Central; 7, West South Central; 8, Mountain; 9, Pacific.

The frequencies of CRE isolates varied widely by Census Division from 0.1% (Census Division 4 [West North Central]) to 2.4% (Census Division 2 [Middle Atlantic]; Supplementary Figure 2). Aztreonam-avibactam retained potent activity against CRE isolates from all Census Divisions (Table 1 and Figure 2). Only 6 CRE isolates (1.4%) exhibited an elevated aztreonam-avibactam MIC (>4 mg/L): 3 *Escherichia coli* isolates 1 each from Census Divisions 2 (Middle Atlantic), 3 (East North Central), and 7 (West South Central), 2 *Klebsiella aerogenes* isolates 1 each from Census Divisions 6 (East South Central) and 7 (West South Central), and 1 *Klebsiella oxytoca* from Census Division 9 (Pacific). In contrast, the activities of other β-lactams against CRE varied widely. CRE susceptibility varied from 67.7% (Census Division 8 [Mountain; n = 31]) to 100.0% (Census Division 4 [West North Central; n = 5]) for ceftazidime-avibactam; from 74.2% (Census Division 8 [Mountain; n = 31]) to 100.0% (Census Division 4 [West North Central; n = 5] and Census Division 6 [East South Central; n = 18]) for meropenem-vaborbactam, and from 71.4% (Census Division 1 [New England; n = 7]) to 100.0% (Census Division 8 [Mountain; n = 31]) for cefiderocol (Table 1 and Figure 2). CRE susceptibility to non–β-lactam compounds also varied markedly by Census Division (Table 1). Notably, aztreonam-avibactam retained activity against Enterobacterales that were nonsusceptible to ceftazidime-avibactam and/or meropenem-vaborbactam (n = 73; MIC_50/90_, 0.25/2 mg/L; 98.6% inhibited at ≤8 mg/L; data not shown).

Results of cross-resistance among new β-lactamase inhibitor combinations are shown in Table 2. Aztreonam-avibactam showed good activity against isolates nonsusceptible to ceftazidime-avibactam (88.4% inhibited at ≤4 mg/L), meropenem-vaborbactam (95.3% inhibited at ≤4 mg/L), and imipenem-relebactam 97.8% inhibited at ≤4 mg/L). Moreover, meropenem-vaborbactam and imipenem-relebactam showed good activity against isolates with aztreonam-avibactam MIC >4 mg/L (83.3% to 84.6% susceptible). In contrast, elevated rates of cross-resistance were observed among ceftazidime-avibactam, meropenem-vaborbactam, and imipenem-relebactam (Table 2).

**Table 2.** Cross-resistance Among Most Recent β-lactamase Inhibitor Combinations

Antimicrobial Agent	% Susceptible Per CLSI or FDA Criteria (No. of Isolates)			
	ATM-AVI MIC >4 mg/L (20)	CAZ-AVI- NS (69)	MEM-VAB- NS (64)	IMI-REL- NS (90)^a^
Aztreonam-avibactam^b^	0.0	88.4	95.3	97.8
Ceftazidime-avibactam	60.0	0.0	31.2	47.8
Meropenem-vaborbactam	83.3	33.8	0.0	40.0
Imipenem-relebactam	84.6	18.0	9.4	0.0

Abbreviations: ATM-AVI, aztreonam-avibactam; CAZ-AVI, ceftazidime-avibactam; FDA, Food and Drug Administration; MEM-VAB, meropenem-vaborbactam; IMI-REL, imipenem-relebactam.

^a^Exclude *P. mirabilis* and indole-positive Proteeae due to intrinsic resistance to this compound.

^b^Percentage of isolates inhibited at ≤4 mg/L of aztreonam-avibactam.

**Figure 3.**
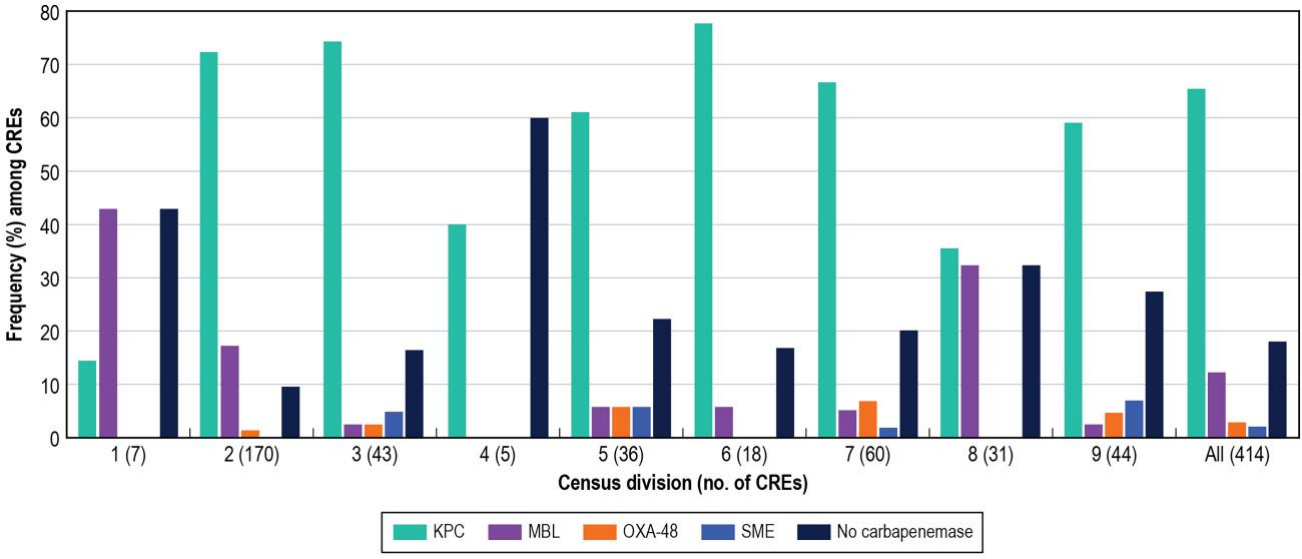
Frequency of carbapenemases among carbapenem-resistant Enterobacterales (CRE) isolates stratified by US Census Division. Census Divisions: 1, New England; 2, Middle Atlantic; 3, East North Central; 4, West North Central; 5, South Atlantic; 6, East South Central; 7, West South Central; 8, Mountain; 9, Pacific.

In general, KPC was the most common carbapenemase (65.5% of CREs, excluding MBL coproducers), followed by NDM (10.6%), OXA-48–like (2.7%; excluding MBL coproducers), and *Serratia marcescens* enzyme (SME; 1.9%; Figure 3). Two carbapenemases were observed in 7 isolates: an NDM and an OXA-48–like in 4 isolates, a KPC and an NDM in 2 isolates, and a KPC and “active in imipenem” in 1 isolate. Overall, an MBL gene was detected in 12.1% of CREs (Figure 3). The occurrence of KPC among CREs varied from 14.3% (1/7 isolates; Census Division 1 [New England]) to 77.8% (14/18 isolates; Census Division 6 [East South Central]), whereas the frequency of MBLs ranged from <3.0% (Census Divisions 3, 4, and 9) to 32.3% (10/31 isolates) in Census Division 8 (Mountain) and as high as 42.9% (3/7 isolates) in Census Division 1 (New England; Figure 3).

The activities of the new β-lactamase inhibitors and cefiderocol against KPC producers varied from 96.3% for imipenem-relebactam to 100.0% for aztreonam-avibactam (Table 3). Only aztreonam-avibactam showed good activity against MBL producers (MIC_50/90_, 0.25/0.5 mg/L; 98.0% inhibited at ≤4 mg/L; Table 3 and Figure 1). Cefiderocol (MIC_50/90_, 2/16 mg/L; 74.0% susceptible) showed moderate activity and the other β-lactamase inhibitor combinations showed very limited activity against these organisms (Table 3). Moreover, only aztreonam-avibactam, ceftazidime-avibactam, and cefiderocol were active against OXA-48–like producers (100.0% susceptibility; Table 3). All SME-producing isolates were susceptible to aztreonam-avibactam, ceftazidime-avibactam, meropenem-vaborbactam, and cefiderocol and resistant to imipenem-relebactam (Table 3).

**Table 3.** Activity of β-lactamase Inhibitor Combinations and Cefiderocol Against CRE Isolates Stratified by Carbapenemase Type

Antimicrobial Agent	% Susceptible Per CLSI or FDA Criteria (No. of Isolates)				
	KPC Producers (271)^a^	MBL Producers (50)	Oxa-48–like- Producers (11)^a^	SME Producers (8)^a^	Non Carbapenemase Producers (74)
Aztreonam-avibactam^b^	100.0	98.0	100.0	100.0	93.2
Ceftazidime-avibactam	98.9	4.0	100.0	100.0	95.9
Meropenem-vaborbactam	98.5	16.0	18.2	100.0	87.8
Imipenem-relebactam	96.3	2.0	9.1	0.0	89.2
Cefiderocol	98.9	74.0	100.0	100.0	89.2

^a^Exclude isolates that co-produce other carbapenemases.

Notably, a carbapenemase gene was not identified in 17.9% of CRE isolates (Figure 3). These isolates (n = 74) were highly susceptible to aztreonam-avibactam (97.3% inhibited at ≤8 mg/L) and ceftazidime-avibactam (95.9% susceptible), and showed moderate susceptibility to meropenem-vaborbactam, imipenem-relebactam, and cefiderocol (87.8% to 89.2% susceptible; data not shown). Higher frequencies of non–carbapenemase-producing CRE were observed in Census Divisions 1 (New England [42.9%; 3/7]), 4 (West North Central [60.0%; 3/5]), 5 (South Atlantic [30.6%; 11/36]), and 8 (Mountain [32.3%; 10/31]) when compared to other Census Divisions (Figure 3). Additionally, among CRE isolates not susceptible to cefiderocol (n = 24), a carbapenemase gene was not identified in 8 isolates (33.3%), whereas the remaining 16 cefiderocol-nonsusceptible isolates had NDM-1 (10 isolates), NDM-1 plus OXA-232 (1), NDM-5 (1), VIM-1 (1), KPC-3 (2), and KPC-4 (1).

## DISCUSSION

The newer β-lactamase inhibitor combinations ceftazidime-avibactam, meropenem-vaborbactam, and imipenem-relebactam as well as the siderophore cephalosporin cefiderocol have become the main options for the treatment of severe infections resulting from CRE [4]. There are major differences in the antimicrobial spectrum of these compounds because of their stability to hydrolysis by the most clinically relevant carbapenemases. Moreover, the dissemination of MBLs, mainly NDM, now represents a new challenge worldwide, including in some US regions because none of the β-lactamase inhibitor combinations listed previously are active against MBL producers and cefiderocol has shown limited activity against some MBLs [8, 9].

Infections involving MBL-producing CRE are associated with markedly higher 30-day mortality when compared to those caused by carbapenem-susceptible isolates, and inappropriate initial antimicrobial therapy certainly plays an important role in these unsatisfactory outcomes [21, 22]. In addition, most MBL producers are also resistant to other antimicrobial classes, further complicating the management of infected patients. Thus, defining and updating the contemporary epidemiology of carbapenemase-producing CRE becomes critical to control the spread of these organisms and to design appropriate regional empiric therapy guidelines, especially for critically ill patients [23, 24].

The results of the present study showed an important variation among US Census Divisions regarding the frequency of CRE as well as the carbapenemase genes carried by these organisms. The occurrence of CREs was much higher in Census Divisions 2 and 7 compared to other Census Divisions. These 2 Census Divisions include the New York City area (Census Division 2) and Texas (Census Division 7), which are known for having medical centers with a high prevalence of CRE, mainly KPC producers [24, 25]. Our results also showed an increasing prevalence of MBLs in some regions, including Census Divisions where the prevalence of CRE was not elevated, like Census Divisions 1 (New England; 42.9% of CREs) and 8 (Mountain; 32.3% of CREs), emphasizing the importance of the implementation of comprehensive surveillance programs that evaluate resistance mechanisms.

A recent increase in the occurrence of MBLs in the United States has been previously reported by our group as well as by other investigators [7, 22]. Lee et al. evaluated the epidemiology of carbapenem-resistant *K pneumoniae* in hospitals across a large public health system in New York City using the National Healthcare Safety Network data [24]. The results of their investigation showed a decline in the frequency of carbapenem-resistant *K pneumoniae* during 2016–2020 followed by a notable increase between January 2021 and June 2022. Lee et al. also observed a marked increase in MBL-producing *K pneumoniae* in the region starting in 2021. Most patients with an MBL originated from long-term care facilities [24].

The results of our investigation indicated that the emergence and increasing frequency of MBLs, mainly NDM, is adversely affecting the spectrum of activity of the most recently approved β-lactamase inhibitor combinations as well as cefiderocol. The activities of these compounds were particularly compromised in Census Divisions with high prevalence of MBLs, such as Census Divisions 1 (New England), 2 (Middle Atlantic), and 8 (Mountain; Figure 3).

It is important to understand the limitations of the study when analyzing its results. That the INFORM Program does not included participant centers in all US states could be considered a limitation; however, at least 3 states were evaluated in each Census Division and the number of isolates per Census Division was significant, ranging from 3294 to 7799. The lack of ertapenem in the criteria used to identify CRE isolates could also be considered a limitation of the study. We observed in previous investigations that isolates resistant to ertapenem and susceptible to both imipenem and meropenem generally display overexpression of AmpC and do not have any carbapenemase gene; however, we understand that we may have missed carbapenemase-producing isolates, especially OXA-48–like producers, by not testing ertapenem. Despite this potential limitation, the results presented here demonstrated that aztreonam-avibactam is highly active against Enterobacterales and its activity is not adversely affected by resistance to β-lactams currently used in clinical practice, including the new β-lactamase inhibitor combinations and cefiderocol. Our results also emphasize the importance of comprehensive surveillance programs to detect regional variations on the epidemiology carbapenemases and other resistance mechanisms.

## Supplementary Material

ofaf005_Supplementary_Data

## Supplementary Data

Supplementary materials are available at *Open Forum Infectious Diseases* online. Consisting of data provided by the authors to benefit the reader, the posted materials are not copyedited and are the sole responsibility of the authors, so questions or comments should be addressed to the corresponding author.

## Notes

***Acknowledgments.*** The authors thank all the participant centers of the INFORM Program for their work in providing isolates.

***Author contributions.*** H.S.S.: Conceptualization, Formal Analysis, Data Curation, Writing—Original Draft, Visualization, Funding Acquisition. J.H.K.: Methodology, Formal Analysis, Investigation, Data Curation, Software, Validation, Supervision. T.B.D.: Methodology, Formal Analysis, Data Curation, Review & Edit, Software, Validation, Supervision. M.L.W.: Conceptualization, Validation, Resources, Supervision, Funding Acquisition. M.C.: Conceptualization, Validation, Resources, Writing—Review & Edit, Supervision, Funding Acquisition. All authors reviewed and approved the manuscript.

***Patient consent statement.*** This study does not include factors necessitating patient consent.

***Financial*  *support.*** This study was supported by AbbVie. Helio S. Sader, John H. Kimbrough, Timothy B. Doyle, Marisa L. Winkler, Mariana Castanheira are employees of Element Iowa City (JMI Laboratories), which was a paid consultant to AbbVie in connection with the development of this poster.
