# Supplementary material for: Frequency, Antimicrobial Susceptibility, and Molecular Characterization of Carbapenem-Resistant Enterobacterales Stratified by United States Census Divisions: Results From the INFORM Program (2018–2022)
Source: Open Forum Infect Dis. 2025 Apr 11;12(4):ofaf005. doi: 10.1093/ofid/ofaf005 (PMC11986335; doi:10.1093/ofid/ofaf005)
Supplement: ofaf005_Supplementary_Data [file ofaf005_supplementary_data.docx]

Figure S1 Map of the United States Census Divisions with the location of INFORM participant medical centers represented with blue dots


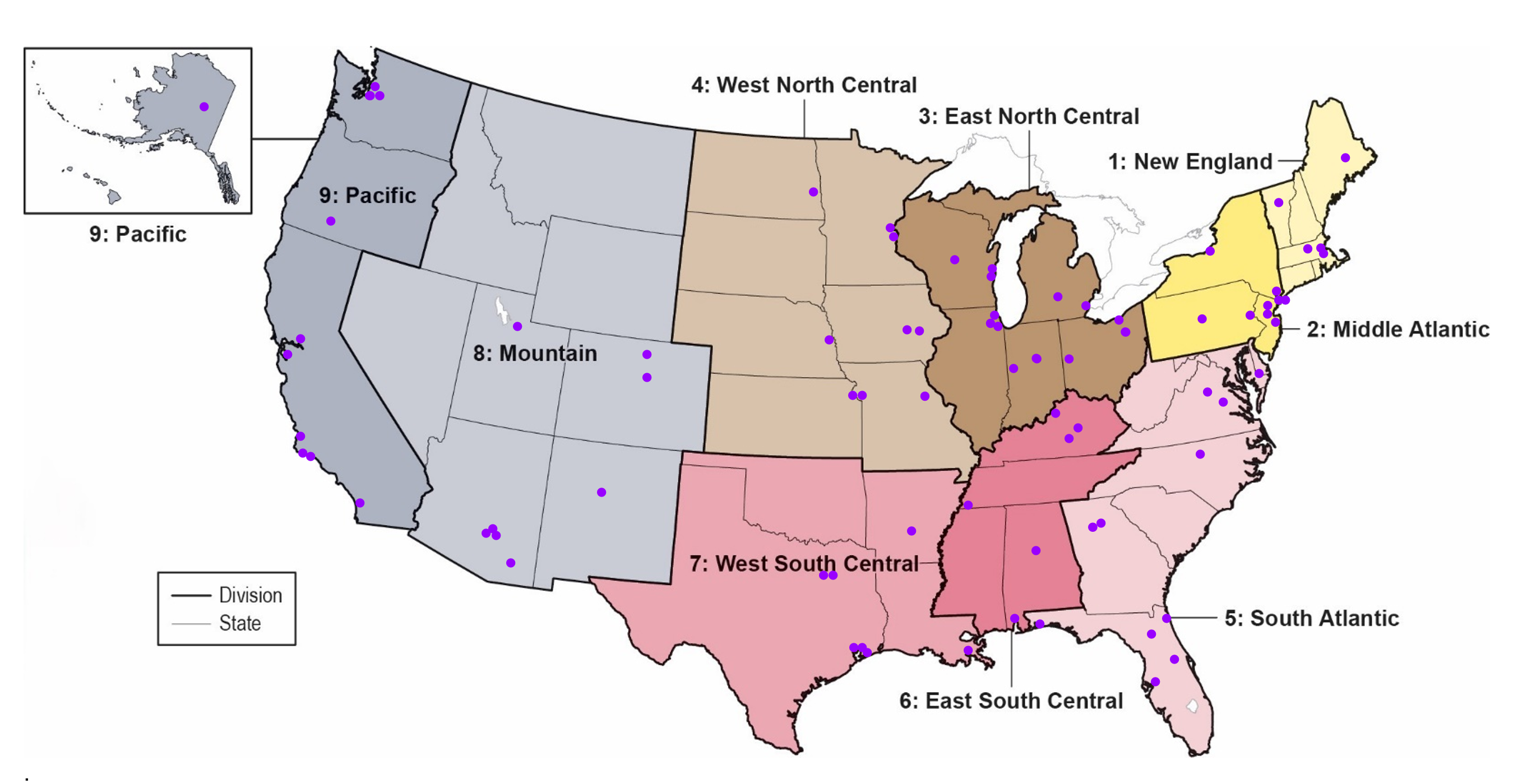


Figure S2 Frequency of carbapenem-resistant Enterobacterales (CRE) stratified by US Census Division (2018–2022)


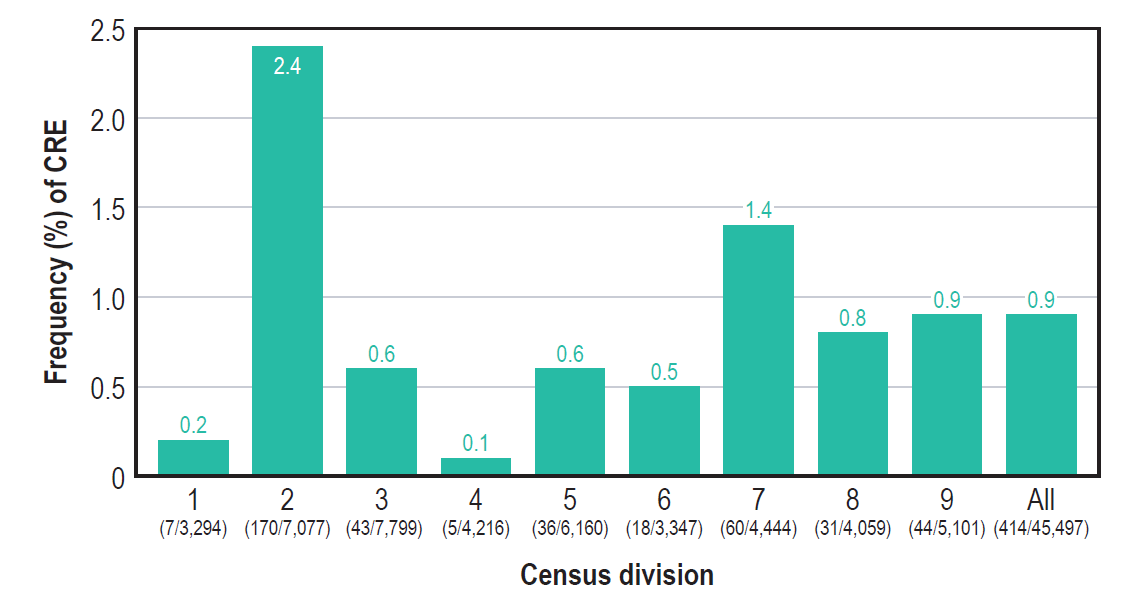


Census Divisions: 1, New England; 2, Middle Atlantic; 3, East North Central; 4, West North Central; 5, South Atlantic; 6, East South Central; 7, West South Central; 8, Mountain; 9, Pacific. Numbers in parentheses indicate the number of CREs (numerator) and total number of Enterobacterales (denominator).
